# A novel deep learning-based method for COVID-19 pneumonia detection from CT images

**DOI:** 10.1186/s12911-022-02022-1

**Published:** 2022-11-02

**Authors:** Ju Luo, Yuhao Sun, Jingshu Chi, Xin Liao, Canxia Xu

**Affiliations:** 1 Third Xiangya Hospital, Central South University, NO.138, Tongzipo Road, Changsha, 410013 Hunan China; 2 College of Computer Science and Electronic Engineering, Hunan University, Changsha, China

**Keywords:** Artificial intelligence, Deep learning, COVID-19, Community acquired pneumonia, CT image

## Abstract

**Background:**

The sensitivity of RT-PCR in diagnosing COVID-19 is only 60–70%, and chest CT plays an indispensable role in the auxiliary diagnosis of COVID-19 pneumonia, but the results of CT imaging are highly dependent on professional radiologists.

**Aims:**

This study aimed to develop a deep learning model to assist radiologists in detecting COVID-19 pneumonia.

**Methods:**

The total study population was 437. The training dataset contained 26,477, 2468, and 8104 CT images of normal, CAP, and COVID-19, respectively. The validation dataset contained 14,076, 1028, and 3376 CT images of normal, CAP, and COVID-19 patients, respectively. The test set included 51 normal cases, 28 CAP patients, and 51 COVID-19 patients. We designed and trained a deep learning model to recognize normal, CAP, and COVID-19 patients based on U-Net and ResNet-50. Moreover, the diagnoses of the deep learning model were compared with different levels of radiologists.

**Results:**

In the test set, the sensitivity of the deep learning model in diagnosing normal cases, CAP, and COVID-19 patients was 98.03%, 89.28%, and 92.15%, respectively. The diagnostic accuracy of the deep learning model was 93.84%. In the validation set, the accuracy was 92.86%, which was better than that of two novice doctors (86.73% and 87.75%) and almost equal to that of two experts (94.90% and 93.88%). The AI model performed significantly better than all four radiologists in terms of time consumption (35 min vs. 75 min, 93 min, 79 min, and 82 min).

**Conclusion:**

The AI model we obtained had strong decision-making ability, which could potentially assist doctors in detecting COVID-19 pneumonia.

## Background

Since the beginning of 2020, the highly contagious novel coronavirus disease 2019 (COVID-19) has spread widely all over the world, causing a tremendous impact on the health and epidemic prevention systems of global countries, claiming millions of lives. After the novel coronavirus infects the human body, it mainly corrodes the lung region and causes lung inflammation, acute respiratory distress, or multiple organ failure in severe cases [1–4].

Reverse transcription polymerase chain reaction (RT-PCR) is used by doctors to evaluate whether patients are infected with novel coronavirus pneumonia (NCP). However, its harsh testing environment inevitably affects the rapid screening of suspected cases. As rapid RT-PCR testing becomes more available, challenges remain, including high false negative rates and the sensitivity sometimes reported as low as 60–70% [5, 6].

As an important supplement to RT-PCR testing, radiographic imaging techniques, such as X-ray examination and computed tomography (CT), similarly play an indispensable role in the auxiliary diagnosis of NCP [7]. CT can detect early COVID-19 in patients with a negative RT-PCR test [8]. During the radiological examination of confirmed cases, researchers found that patients without symptoms, or before patients develop symptoms or after symptoms resolved, chest X-rays and CT images already showed changes associated with pneumonia induced lesions [9–11].

Artificial intelligence (AI) has profoundly transformed the way we live our lives, especially in the field of medical science. Deep learning (DL), a branch of AI, has made tremendous progress in diagnosis assistance and prognosis prediction along with the accumulation of abundant medical data and the improvement of computer algorithms. During the pandemic, due to the rapid increase in the number of new and suspected COVID-19 cases, DL had the potential to aid in the rapid evaluation of CT scans for the differentiation of COVID-19 findings from other clinical problems.

Some studies have already demonstrated the potential for AI-based diagnosis. Harmon et al. utilized a series of deep learning algorithms trained in a diverse multinational cohort of 1280 patients to detect COVID-19 pneumonia and achieved up to 90.8% accuracy [12]. Zhang et al. developed an AI system that can diagnose NCP and differentiate it from other common pneumonia and normal controls [13]. Fang et al. developed an early-warning system with deep learning techniques to predict COVID-19 malignant progression [14]. Yildirim et al. proposed a hybrid approach for diagnosing COVID-19 on chest X-ray images. The accuracy values obtained in the two different datasets were 99.05 and 97.1%, respectively [15]. Another study developed a hybrid model to diagnose COVID-19 from X-ray images and compared it with AlexNet, Resnet50, GoogLeNet, and VGG16. This hybrid model achieved the highest accuracy [16].

To assist physicians and radiologists in improving the accuracy of diagnosis and relieving the fatigue of checking large CT images. This research focused on the identification of COVID-19 infections in lung CT images in open source multi-institute and multidisease datasets. And propose a novel deep learning-based method for COVID-19 detection from CT images that achieves state-of-the-art accuracy.

## Methods

### Study population

This retrospective study included 222 patients positive for COVID-19, 88 community-acquired pneumonia (CAP), and 127 normal cases. Each patient contained 150–200 CT images provided with the Digital Imaging and Communications in Medicine (DICOM) format. All patients and CT images were obtained from the 2021 IEEE ICASSP Signal Processing Grand Challenge. All CT scans in training and validation were obtained by a SIEMENS SOMATOM Scope scanner with a normal radiation dose and a slice thickness of 2 mm. The test data-set was obtained from a different medical center with various slice thicknesses and radiation doses. COVID-19 cases were collected from February 2020 to April 2020, whereas CAP cases and normal cases were collected from April 2018 to December 2019 and January 2019 to May 2020, respectively. The diagnosis of COVID-19 infection was based on positive RT-PCR test results, clinical parameters, and CT scan manifestations identified by three experienced thoracic radiologists. The diagnosis of CAP was based on clinical parameters and CT scan manifestations identified by three experienced thoracic radiologists.

### CT images datasets

The study population was divided into training, validation, and test datasets. Patient labels provided by three radiologists showed a high degree of agreement (more than 90%). Based on the patient-level labels, we built a slice-level labeled CT image data-set. Every case was analyzed by the radiologist to identify and label slices with evidence of infection. The labeled CT-image data-set contained 14,976 slices demonstrating infection and 40,553 slices without infection. The data-set was then divided into training, validation, and test sets, which are described in Table 1.

**Table 1:** The information of all datasets

	Training datasets (patients/slices/infection slices)	Validation datasets (patients/slices/infection slices)	Test datasets (patients)
Normal	52/9321/0	24/4525/0	51
CAP	41/7520/2468	19/3451/1028	28
COVID-19	116/20,208/8104	55/10,504/3376	51

### Data preprocessing

Data preprocessing mainly included lung region extraction and format conversion of slice images. U-Net with batch normalization after each layer as an automatic lung segmentation model for image preprocessing. The U-Net model performs precise segmentation on individual slices and extracts the right-left lung separately, including air pockets, tumors, and effusions. Therefore, helping the classification network focus more on the association between ground glass shadows and slice lesions. In the data-set, slices were displayed in DICOM format. To better adapt to the network, this study carried out a format conversion. The converted PNG format images were sorted according to the slice location value in the DICOM format to ensure that slices and labels corresponded to each other. The conversion threshold was adjusted according to the lung window range so that the lung tissue in the image was displayed more clearly and the lung texture highlighted.

### Classification model

**Fig. 1:**
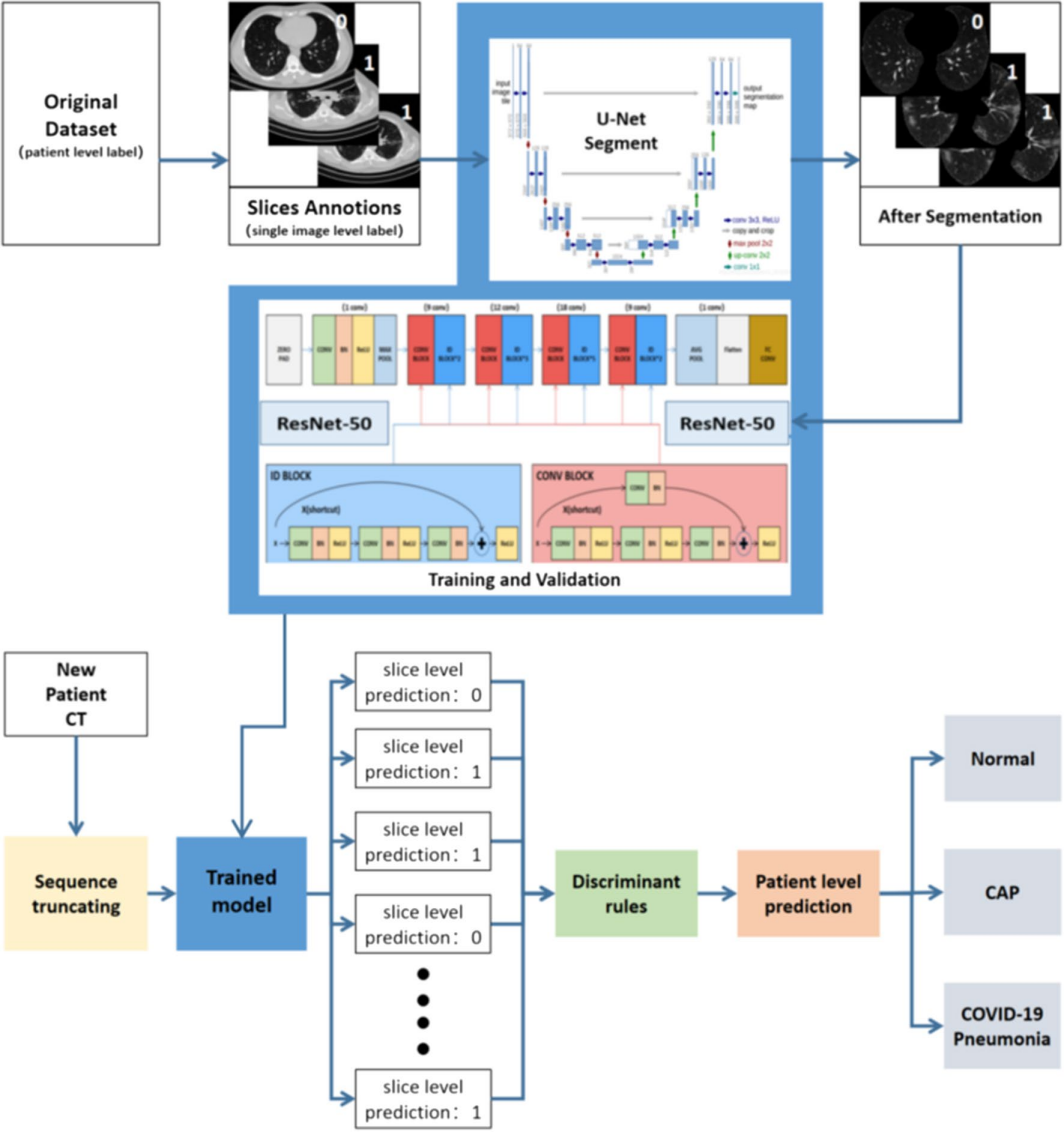
The architecture of proposed solution

Classification and discrimination algorithms mainly included backbone network training based on ResNet-50 and custom discrimination rules based on clinical diagnosis experience. In order to prevent over-fitting, the Batch-Normalization layer, decay learning rate method and early stop strategy work together to inhibit over-fitting. In the model training phase, we focused on the processing of input data. Through the observation of the images after lung segmentation, it was found that the first 10% and the last 14% part of the patient slice sequence rarely included image semantic information that was associated with distinguishing features. Experiments show that the classification accuracy of the network trained after truncating the patient slice set can be significantly improved. After the slice sequence was truncated at a certain ratio at the head and tail, the patient-level symptoms of the current sequence were classified according to the relative number of remaining slice prediction categories. When the current sequence was judged to be COVID-19 or CAP, the system directly output the prediction result. When the sequence was classified as normal, the system again compared the quantity of COVID-19 and CAP slices in the sequence. If their number exceeded 20% of the sequence length, the sequence was further judged as COVID-19 or CAP according to the relative quantity of corresponding slices. Figure 1 shows the architecture of proposed method. Relevant codes and models can be freely accessed at https://github.com/philiplaw1984/COVID-19/.

### AI vs. doctors

A radiologist with different qualifications, including two experts and two novices, was defined as a radiologist who had clinical experience of two years or less, and an expert was defined as a radiologist who had clinical experience of five years or more. All four radiologists were asked to classify the data of the same validation set, and their results were compared with the AI model. The accuracy in classifying normal, CAP, and COVID-19 patients were calculated. The sensitivity of identifying COVID-19 patients and time consumption of diagnosis for the entire validation set were also calculated.

## Results

### Ablation comparison results in the validation dataset

For the model evaluation, 98 patient’s slice sequences were divided into validation dataset, including 55 COVID-19 cases, 19 CAP cases, and 24 normal cases. Experiments showed that the classification accuracy of the network trained after truncating the patient slice set or introduce customized rules can be significantly improved. The results are shown in Table 2.

**Table 2:** Comparison of ablation experiment results in three classification tasks

Ablation comparison	Sens (C1)	Sens (C2)	Sens (C3)	Acc
Without sequence truncating	0	0.7895	0.8727	0.6429
Without customized rules	1	0.3158	0.4909	0.5816
Proposed method	1	0.7895	0.9455	0.9286

“C1” represents “Normal”, “C2” represents “CAP”, and “C3” represents “COVID-19”

### Classification model performance in the validation dataset

**Fig. 2:**
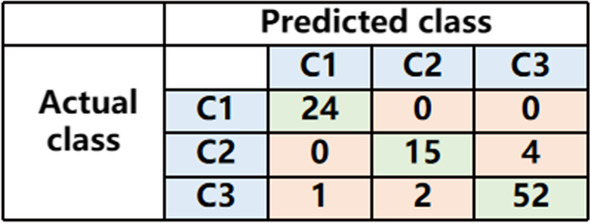
The confusion matrix for the three-category classification task in the validation dataset

For the three-category classification task, Fig. 2 shows the confusion matrix. In the validation dataset, the accuracy of was 92.86%. In addition, The sensitivity for detecting COVID-19 were calculated. The sensitivity was 94.55%. The total diagnosis time of all 98 cases in the validation set was 35 min. The other results are shown in Table 3.

**Table 3:** Evaluation metrics of the model

	Acc	Sens	Spe	Fpr	Fnr	Fdr	F1
Normal	0.9898	1	0.9865	0.0135	0	0.04	0.9796
CAP	0.9388	0.7895	0.9747	0.0253	0.2105	0.1176	0.8334
COVID-19	0.9286	0.9636	0.907	0.093	0.0364	0.0714	0.9458

### Radiologist performance in the validation dataset

**Fig. 3:**
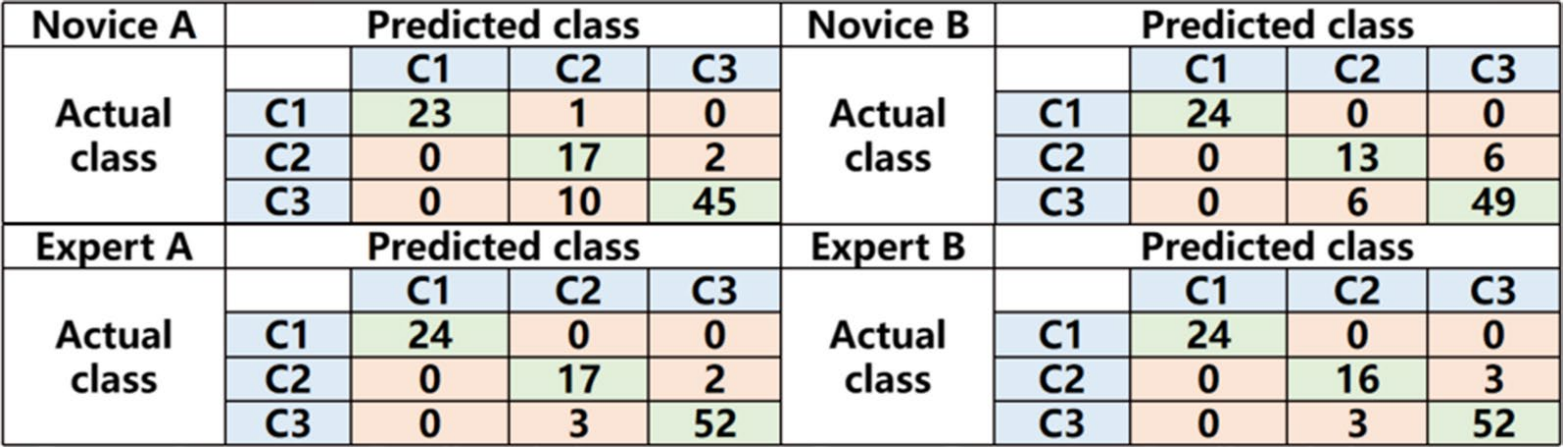
The confusion matrix for the three-category classification task in the validation set of radiologists

For the three-category classification task, Fig. 3 shows the confusion matrix. In the validation dataset, the accuracy of the two novice radiologists was 86.73% and 87.75%. The accuracy of the two expert radiologists was 94.90% and 93.88%. The sensitivity of the two novice radiologists for identifying COVID-19 was 81.82% and 89.09%. The sensitivity of both expert radiologists for identifying COVID-19 was 94.55%. The total diagnosis time of the two novice radiologists was 75 min and 93 min, and the total diagnosis time of the two expert radiologists was 79 min and 82 min.

### AI vs. doctors

**Fig. 4:**
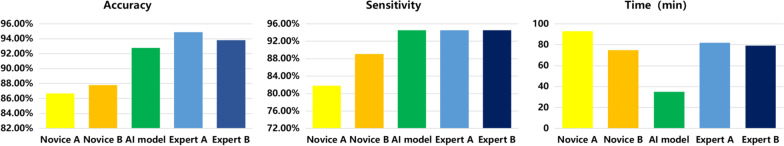
The performance of AI versus radiologists

In the validation set, the diagnostic accuracy of AI model for all three classifications was 92.86%. The sensitivity of the model for COVID-19 was 94.55%. The results indicated that AI model performed significantly better than all four radiologists in terms of time consumption. The AI model performed better than novice radiologists and better than expert radiologists in terms of the accuracy and sensitivity of diagnosis, as shown in Fig. 4.

### Classification model performance in the test set

**Fig. 5:**
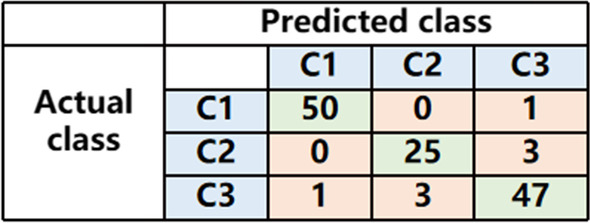
The confusion matrix for the three-category classification task in the test set

For the three-category classification task, Fig. 5 shows the confusion matrix. In the test dataset, the accuracy was 93.84%. In addition, we calculated the sensitivity of the three classes. The sensitivity (C1) was 0.9803, the sensitivity (C2) was 0.8928, and the sensitivity (C3) was 0.9215.

The experiments demonstrated that the proposed solution was effectively utilized to solve the classification problem of COVID-19, CAP, and normal cases based on lung CT images. All the datasets in this study were obtained from the 2021 IEEE ICASSP Signal Processing Grand Challenge. Our proposed method was the first place winner of this challenge.

## Discussion

In this study, an AI system for the diagnosis of COVID-19 pneumonia based on ResNet-50 and U-Net were developed. U-Net is an automatic lung segmentation method based on the semantic segmentation architecture, which will help the network focus on the lung region and extract distinguishing features [17]. Lung segmentation is able to extract all the lung regions, thereby helping the classification network focus more on the association between ground glass shadows and solid lesions. Then, the ResNet-50 was used for every CT slice classification. ResNet-50, which is often used as the backbone of the classification network, is also widely used in COVID-19 detection tasks [18]. Çınar et al. developed a model based on the layers of ResNet50. With this new model, the diagnosis of pneumonia can be made early and accurately [19]. Narin et al. [20] used ResNet-50 to solve the detection of novel coronavirus infection in chest X-ray images and obtained satisfactory accuracy. Aram et al. [21] used ResNet-50 as the feature extractor in the training phase, and in the COVNet proposed by Li et al. [22], parallel ResNet-50 with shared weights constituted their model.

However, the AI system is often hampered by problems with low universality due to the uniformity of data. Therefore, our study was specifically designed to maximize the potential for generalizability. All the CT images come from an open source dataset collected by multiple institutes in Canada. The training and validation datasets have a normal radiation dose and a slice thickness of 2 mm. The CT images from the test dataset were obtained from different medical centers with various exposure doses, slice thicknesses, and ranges of values in the Hounsfield Unit. Surprisingly, AI system’s performance in the test dataset was equally matched with the validation dataset. Thus it can be seen that our AI system has excellent universality and robustness.

Several factors set this study apart from similar prior efforts: (1) to expand the training images, we performed additional slice-level annotations on all patient slice sequences marked as “Training” the dataset. (2) The U-Net-based semantic segmentation architecture was used to segment each lung slice. Only the lung tissue, not the original CT images, was input into the network for training. (3) We chose the ResNet-50 model, which is commonly used in COVID-19 detection, as the backbone of the classification network. However, we defined the discriminant rules based on the clinical diagnosis and treatment experience of medical imaging. Recently, most AI and COVID-19 studies have focused on big data and complex computer algorithms. Few studies have introduced discriminant rules into the AI model.

RT-PCR from respiratory samples is the standard for diagnosis. However, the sensitivity of testing varies from 33 to 80% [23–25]. Chest CT imaging findings are nonspecific and overlap with other infections, especially CAP, so the diagnostic value of CT imaging for COVID-19 is limited [26]. However, with the help of the AI model, doctors could distinguish CAP and COVID-19 more easily. Moreover, under some circumstances, patients admitted to the hospital with abnormal chest CT imaging findings compatible with RT-PCR were negative. If the model was highly accurate for COVID-19, doctors could increase the frequency of RT-PCR tests and transfer and treat patients earlier. In that way, it would be of great help to the patient and public health.

Our study carried out an AI versus radiologist experiment. The results showed that the AI model detected COVID-19 with a high accuracy of 93.84% and sensitivity of 92.15%, which was better than novice radiologists and equivalent to expert radiologists. This indicates that the AI model could significantly improve the ability of novice radiologists. In addition to diagnostic accuracy and sensitivity, this study also found that the AI method showed a significant saving of time compared to all radiologists. When the COVID-19 outbreak occurred, many patients rushed to the hospital, and a massive number of suspected patients needed to be excluded. COVID-19 has the potential to overwhelm the local health care system. However, with the assistance of the AI model, COVID-19 could be diagnosed quickly and accurately. The AI model will provide strong support to the medical system, especially in areas with weak medical infrastructure.

There are several limitations to this study. First, the sample size of our research was smaller than that of other multinational studies. All the patients and CT images were not collected by our own but from an open source dataset released by the 2021 IEEE International Conference on Acoustics, Speech and Signal Processing. Second, all COVID-19 cases in this study were based on RT-PCR positivity and CT positivity. However, CT images are often negative despite a positive RT-PCR test [27]. The AI model may not have the ability to identify CT-negative cases. However, more suitable for the situation when the RT-PCR is negative but patient is highly suspected to be infected. If the AI model was diagnosed with COVID-19, repetitive RT-PCR is indispensable. Finally, the AI algorithm aimed to classify normal, CAP, and COVID-19 pneumonia cases. However, many other pneumonias, such as fungal infection and influenza pneumonia, also need to be distinguished. The situation in real clinical practice may be more complicated, so the AI model also needs to be improved.

In conclusion, we have seen broad prospects for the combination of deep neural networks and the field of medical imaging. Relying on its powerful feature extraction capability, deep neural networks can obtain excellent discrimination accuracy with the support of sufficient data and appropriate models. Moreover, the customized discriminant rules based on clinical diagnosis experience can impose constraints on the model and improve the interpretability. Combining the above two points, the model this study obtained could have a stronger decision-making ability, which could potentially assist doctors in detecting COVID-19 pneumonia.

## Data Availability

The data that support the findings of this study are available from the 2021 IEEE ICASSP Signal Processing Grand Challenge (SPGC), but restrictions apply to the availability of these data, which were used under license for the current study and are not publicly available. Data are, however, available from the authors upon reasonable request and with permission of the 2021 IEEE ICASSP Signal Processing Grand Challenge (SPGC). All the relevant codes and models can be freely accessed at https://github.com/philiplaw1984/COVID-19/.

## Abbreviations

COVID-19: Coronavirus disease 2019
RT-PCR: Reverse transcription polymerase chain reaction
NCP: Novel coronavirus pneumonia
CT: Computer tomography
AI: Artificial intelligence
DL: Deep learning
CAP: Community acquired pneumonia
DICOM: Digital Imaging and Communications in Medicine
